# Detection of systemic immunosuppressants in autologous serum eye drops (ASED) in patients with severe chronic ocular graft versus host disease

**DOI:** 10.1007/s00417-020-04865-8

**Published:** 2020-08-19

**Authors:** Volkan Tahmaz, Martin H. J. Wiesen, Uta Gehlsen, Laura Sauerbier, Michael E. Stern, Udo Holtick, Birgit Gathof, Christof Scheid, Carsten Müller, Philipp Steven

**Affiliations:** 1 Division for Dry-eye disease and ocular GvHD, Department of Ophthalmology, University of Cologne Medical Faculty and University Hospital of Cologne, Cologne, Germany; 2 Cluster of Excellence: Cellular Stress Responses in Aging-associated Diseases (CECAD), University of Cologne, Cologne, Germany; 3 Center of Pharmacology, Department of Therapeutic Drug Monitoring, University Hospital of Cologne, Cologne, Germany; 4 ImmunEyez LLC., Irvine, CA USA; 5 Department of Internal Medicine I, University Hospital Cologne, Cologne, Germany; 6 Institute of Transfusion Medicine, University of Cologne, Cologne, Germany

**Keywords:** Graft-versus-host disease, Autologous serum eye drops, Immunosuppression; mass spectrometry

## Abstract

**Purpose:**

Chronic graft versus host disease is a major consequence after allogeneic stem cell transplantation (allo-SCT) and has great impact on patients’ morbidity and mortality. Besides the skin, liver, and intestines, the eyes are most commonly affected, manifesting as severe ocular surface disease. Treatment protocols include topical steroids, cyclosporine, tacrolimus, and ASED. Since these patients often receive systemic immunosuppressant therapy from their oncologists, a topical re-administration of these drugs via ASED with potentially beneficial or harmful effects is possible. The purpose of the study was to determine whether and to which extent systemic immunosuppressants are detectable in ASED.

**Methods:**

A total of 34 samples of ASED from 16 patients with hemato-oncological malignancies after allo-SCT were collected during the manufacturing process and screened for levels of cyclosporine, mycophenolic acid, everolimus, and tacrolimus via liquid chromatography coupled with tandem mass spectrometry (LC-MS/MS). The study followed the tenets of the Declaration of Helsinki and informed consent was obtained from the subjects after explanation of the nature and possible consequences of the study.

**Results:**

Cyclosporine was found in 18 ASED samples in concentrations ranging from 6.5–105.0 ng/ml (32.0 ± 22.8 ng/ml, mean ± SD). The concentration range of mycophenolic acid in 19 samples was 0.04–25.0 mg/l (4.0 ± 5.4 mg/l, mean ± SD). Everolimus and tacrolimus concentrations were well below the respective limits of quantification (< 0.6 and < 0.5 ng/ml) of the established LC-MS/MS method in all samples.

**Conclusions:**

Our study suggests that orally administered cyclosporine and mycophenolic acid for the treatment of systemic GvHD, but not everolimus and tacrolimus, are distinctly detectable in ASED in relevant concentrations. It is highly likely that these agents affect topical therapy of ocular GvHD. However, the extent of this effect needs to be evaluated in further studies.

**Electronic supplementary material:**

The online version of this article (10.1007/s00417-020-04865-8) contains supplementary material, which is available to authorized users.

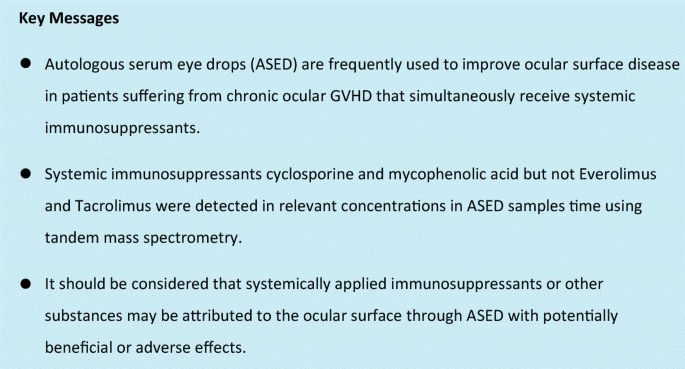



## Introduction

Graft versus host disease (GvHD) is a major complication following allogeneic hematopoietic stem cell transplantation (allo-SCT) and related to immunological reactions mainly mediated by donor CD3+ T cells of the CD8+ subset and directed against host tissues [1]. In general, GvHD develops in three stages: the first phase begins with an activation of antigen-presenting cells (APC) in the host tissue by the underlying disease and the conditioning regime, followed by an activation of transplanted donor T cells leading to the second phase, characterized by cellular response, and finally the third phase, namely the inflammatory effector phase [2]. Historically, GvHD manifesting during the first 100 days after SCT was considered acute GvHD, while a manifestation after the first 100 days post-SCT was diagnosed as chronic GvHD [3]. Since this did not fully capture the distinct clinical differences of the disease entities, the diagnosis of either acute or chronic GvHD is now not only merely reliant on time of onset but also includes clinical feature characteristic for one or the other entity. This made further differentiation necessary and led to the addition of late-onset acute GvHD and overlap syndromes. Incidence is reported to be 40–50% for acute GvHD, depending on the type of stem cell transplantation performed, while 30–70% of patients after 100 days will develop chronic GvHD [2].

Ocular manifestation itself can again be separated into acute and chronic ocular GvHD, with acute ocular GvHD presenting mainly pseudomembranous attachments on and fibrosis of the conjunctiva, whereas chronic ocular GvHD manifests with new-onset dry, gritty or painful eyes, cicatricial conjunctivitis, keratoconjunctivitis sicca, and/or confluent areas of punctate keratopathy [4]. Symptoms include ocular pain, burning sensation, foreign body sensation, and light sensitivity; clinical signs include punctate and filamentary keratopathy, also fibrotic and inflammatory changes in the conjunctiva and blepharitis [5]. For the most part, ocular GvHD is a severe immune-mediated disease of the ocular surface and lacrimal organs with damage inflicted to ocular tissues by inflammation and fibrosis [6]. Severe cases can lead to corneal perforation with necessity of keratoplasty, which itself is associated with a higher risk of transplant failure in patients with GvHD, making it an eyesight-threatening disease entity [7].

Therapy regimes of chronic ocular GvHD are comparable with those of severe dry eye disease, initially based on preservative-free lubricant eye drops and ointments to stabilize the tear film and reduce tear film evaporation. To improve the secretion of the Meibomian glands, patients should clean the lid margins with warm and moist cleaning pads. The heightened inflammatory activity of the ocular surface is initially treated with topical corticosteroids. Reduction of inflammation over longer periods of time by topical application of cyclosporine allows to forgo long-term application of steroids which reduces the risk of steroid-induced side effects including the rise of ocular pressure and accelerated emergence of cataract. Alternatively, tacrolimus can be used as an ointment on the lids [8]. In more severe cases of ocular GvHD, an improvement of visual acuity and improvement of symptoms can be achieved by prescribing scleral contact lenses [9] or autologous serum eye drops (ASED) [10]. ASED have been used for decades to treat severe cases of dry eye–related conditions like neurotrophic keratopathy, Sjögren’s syndrome, or after severe chemical burn [11] and have also become a viable treatment for severe ocular GVHD [12]. The main advantage of ASED in comparison with other treatment options is their effect on all three mechanisms of severe dry eye by lubricating the ocular surface, reducing inflammation, and promoting wound healing, the latter mechanism not being addressed by topical steroids or immunosuppressants. In a recently published retrospective analysis of our patients, we could show a significant improvement of visual acuity, corneal staining, and ocular surface disease index (OSDI) without any reported side effects using 100% autologous serum [13]. For the manufacturing process, a sealed manufacturing system is used in our clinic to deliver the patient’s serum into single-dose containers [13]. However, it should be taken into consideration that patients suffering from GvHD present a medically challenging population, for they often receive extensive systemic therapies with immunosuppressants, antibiotics, and sometimes antiviral and antifungal agents. Although it is highly likely that systemically administered agents will be detectable in eye drops manufactured from patient blood, there have not been any investigations regarding the extent in which specific substances from patient blood are also present in serum eye drops and whether they affect topical therapy positively, negatively, or not at all.

Therefore, the aim of this study was to investigate the presence of systemically applied immunosuppressants in ASED from patients with chronic ocular GvHD and, if possible, correlate the results with clinical courses.

To achieve this, we developed and validated a liquid chromatography coupled with tandem mass spectrometry (LC-MS/MS) method to detect and quantify possible residues of selected immunosuppressants (cyclosporine, tacrolimus, and everolimus) in ASED. For mycophenolic acid, we used a previously published selective and sensitive LC-MS/MS method [14].

## Methods

### Patients

A total of 34 batches of ASED from 16 patients dating between January 2013 and June 2015 were analyzed. In cases where multiple batches from the same patient were analyzed, those batches had been collected during different therapy cycles at different points in time. The eye drops were obtained from residual tubing of the “closed” manufacturing system that is routinely stored in the Institute of Transfusion Medicine for quality control. These samples had been stored in a − 80 °C freezer until analysis. All patients had undergone allogeneic hematopoietic stem cell transplantation due to hematological malignancies (Table 1) and were diagnosed with chronic ocular graft versus host disease NIH grade 3. Systemic immunosuppressive therapy regimes are summarized in Table 2. Clinical evaluation included best corrected visual acuity (BCVA), slit lamp examination including fluorescein staining of the cornea, and evaluation of discomfort via ocular surface disease index (OSDI [15]) among other parameters.

**Table 1:** Distribution of hematological malignancies among included patients

Disease entity	Number of patients (*n* = 16)
Acute myeloid leukemia	7
Acute lymphoblastic leukemia	3
Chronic lymphoblastic leukemia	2
Myeloproliferative neoplasm	2
Hodgkin’s lymphoma	2

**Table 2:** Distribution of systemically administered immunosuppressive drugs among included patients

Immunosuppressants	Number of batches (*n* = 34)
Cyclosporine	7
Mycophenolic acid	5
Cyclosporine and mycophenolic acid	11
Tacrolimus and mycophenolic acid	3
Everolimus	2
No immunosuppressants	6
Total samples	34

### Manufacturing of autologous serum eye drops

ASED were manufactured at the Institute of Transfusion Medicine, University Hospital of Cologne, using a “closed” manufacturing system as previously described [16]. Briefly, the manufacturing process consisted of extracting full-blood from the patient (standard volume approximately 500 ml, depending on the patient’s general health condition), separating the serum via centrifugation and aseptically bottling the serum in vials for freezing and following microbiological testing before distribution to patients through local pharmacies. Residual serum samples within the production system were labeled and stored at − 80 °C until analysis. Patient serum was not diluted during manufacturing, and therapy was conducted with 100% autologous serum eye drops.

### Chemicals and reagents

For LC-MS/MS method development and measurement of immunosuppressant drugs, pure substances of mycophenolic acid, cyclosporine, tacrolimus, and everolimus were purchased from Molekula (Munich, Germany). Mycophenolic acid (MPA) was purchased from Sigma-Aldrich (Steinheim, Germany). The stable isotope-labeled analogues [^13^C,^2^H_3_]-mycophenolic acid, [^2^H_12_]-cyclosporine A, [^13^C,^2^H_2_]-tacrolimus, and [^13^C_2_,^2^H_4_]-everolimus were obtained from Alsachim (Illkirch-Graffenstaden, France). Acetonitrile and formic acid in LC-MS standard quality were purchased from Merck (Darmstadt, Germany). Deionized water was purified with a Milli-Q Plus Ultrapure water system (Millipore Corporation, Bedford, MA, USA).

### Stock solutions, calibration standards, and quality controls

Stock solutions of cyclosporine (139.7 mg/l), tacrolimus (119.0 mg/l), and everolimus (135.4 mg/l) were prepared in methanol and stored at − 80 °C. For the preparation of calibration standards (CS) and quality controls (QC), working stock solutions containing cyclosporine, tacrolimus, and everolimus were arranged. Blank serum samples (provided by the Department of Transfusion Medicine, University Hospital of Cologne) were spiked to obtain six CS (final concentrations: 10, 40, 120, 250, 400, 600 ng/ml (cyclosporine); 1, 5, 10, 20, 30, 50 ng/ml (tacrolimus, everolimus)) and three QC (final concentrations: 20, 200, 500 ng/ml (cyclosporine); 3, 15, 40 ng/ml (tacrolimus, everolimus). Different lots of blank plasma samples were used for the preparation of CS and IQC. CS and QC were freshly spiked for individual validation experiments and LC-MS/MS measurements of ASED samples. A mixture of all internal standards was prepared in acetonitrile.

### Liquid chromatography tandem mass spectrometry

All samples were analyzed using a TSQ Vantage triple-stage quadrupole mass spectrometer (ThermoFisher Scientific, San Jose, CA, USA), working in selected reaction monitoring mode with positive electrospray ionization. The system was equipped with an Accela 1250 pump and an Accela autosampler, fitted with a temperated tray and column oven. The Thermo Xcalibur software (version 2.1) was used for instrument control and data acquisition. The MS/MS conditions were optimized using the Thermo TSQ Tune Master software (version 2.3).

For extraction, samples were admixed with 50-μl acetonitrile containing the isotopically labeled internal standards and 100-μl acetonitrile. The mixture was thoroughly vortexed and centrifuged (10 min, 4 °C, 15,000 g). The clear supernatant was subsequently transferred to LC-MS glass vials (Macherey-Nagel, Düren, Germany). Injection volume was set to 5 μl, and chromatographic separation was achieved using a Hypersil Gold column Hypersil Gold C18 column (50 mm × 2.1 mm, 1.9 m; Thermo Scientific). The mobile phase consisted of 10 mM ammonium formate, 0.05% formic acid in water (A), and 10 mM ammonium formate, 0.05% formic acid in methanol (B). A gradient elution at a flow rate of 300 μl/min was used: 0–1 min 50% A, 1–2 min linear to 10% A, 2–3.5 min linear to 5% A, 3.5–4 min linear to 50% A, and 4–5 min 50% A. MPA concentrations were measured using a previously established LC-MS/MS method with minor modifications [14].

For calibration, the peak area ratios of analytes to respective internal standard areas were plotted against specified concentrations (*x*-axis). Calibration curves were generated by least squares linear regression with a weighting factor of 1/*x*. Linearity was accepted if the linear regression procedure gained *R*^2^ > 0.99. For cyclosporine, tacrolimus, and everolimus, precision and trueness were assessed for all QC levels as inter-day variability (over time on six different days) and intra-day variability (six replicates per concentration analyzed on 1 day). Precision was expressed as the coefficient of variation (CV), calculated as the standard deviation of the observed concentrations divided by the mean concentration for each QC level. Trueness was calculated as the deviation of the mean from the nominal concentration for each QC level. Precision and trueness were accepted if the acceptance criterion ± 15% was not exceeded. Limits of detection (LOD) and limits of quantification (LOQ) were calculated from inter-day assay data, using the equations LOD = 3.3 *σ*/*S*′ and LOQ = 10 *σ*/*S*′, where *σ* is the standard deviation of the blank response and *S*′ is the slope of the calibration curve.

### Clinical data

Data from clinical examinations were extracted from our established database (University Hospital of Cologne, ethical committee approval # 16-405) and screened for efficacy and safety issues during the time course of application of ASED samples analyzed in this study. A total of 16 patients were observed, of whom 34 blood samples were tested for immunosuppressant agents.

### Statistics

Statistical analysis was performed according to the Gaussian distribution of data using the non-parametric Wilcoxon test or paired Student’s *t* test. A *p* value below 0.05 was considered to be significant. All analyses were performed using SPSS vs. 25 (IBM). Statistical analysis was conducted for all patients and the subgroups of immunosuppressant-positive patients (*n* = 12) and immunosuppressant-negative patients (*n* = 4), respectively.

## Results

### Liquid chromatography tandem mass spectrometry

Cyclosporine, tacrolimus, and everolimus eluted within a run time of 5.0 min, and internal standards co-eluted with analytes (Supplemental Fig. 1: chromatograms). No interfering peaks were observed. SRM transitions were as follows: cyclosporine, m/z 1202.9 → 425.3; [^2^H_12_]-cyclosporine A, m/z 1214.9 → 437.2; Tac, m/z 821.5 → 768.6; [^13^C, ^2^H_2_]-tacrolimus, m/z 824.5 → 771.6; Eve, m/z m/z 975.6 → 908.7; [^13^C_2_, ^2^H_4_]-everolimus, m/z 981.6 → 914.7.

Linear quantification was achieved for all analytes (*R*^2^ > 0.996). Exemplary calibration curves are shown in Supplemental Fig. 2: calibration curves. Inter- and intra-day precisions and trueness remained well within the acceptance criterion of ± 15%. The calculated LOD were 0.5 ng/ml for cyclosporine and 0.2 ng/ml for both tacrolimus and everolimus. The calculated LOQ were 1.6 ng/ml, 0.5 ng/ml, and 0.6 ng/ml for cyclosporine, tacrolimus, and everolimus, respectively. Method specifications for MPA were previously described [14].

### Concentrations of immunosuppressants in ASED

**Fig. 1:**
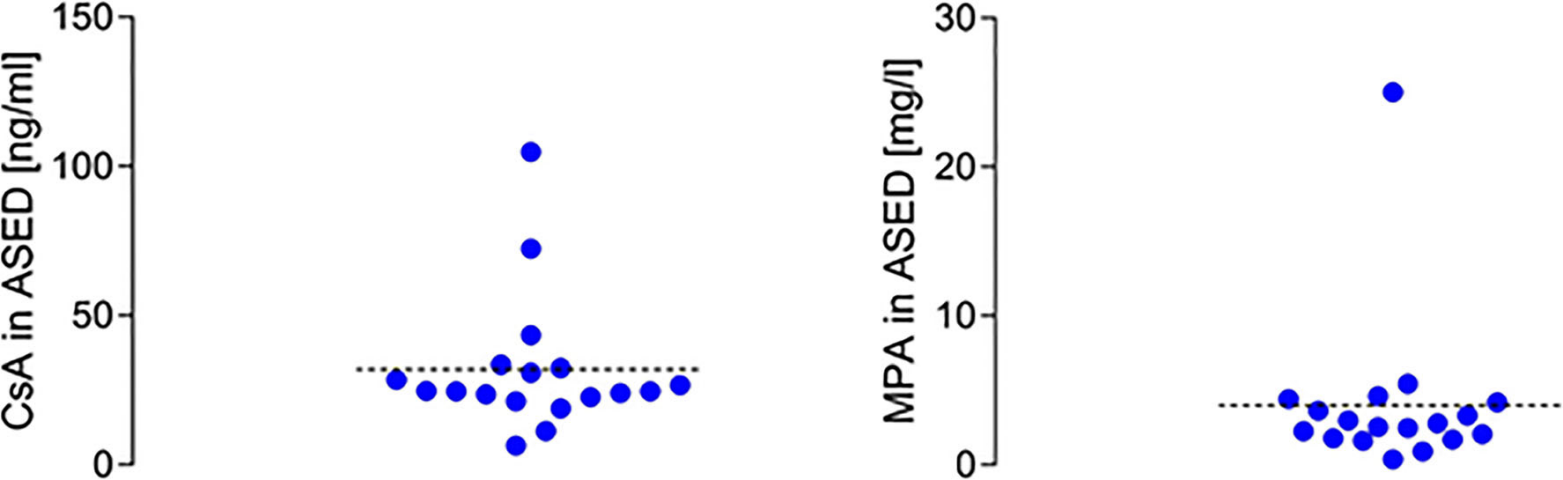
Distribution of observed cyclosporine (Csa) and mycophenolic acid (MPA) ASED concentrations

Sixteen patients with hemato-oncological malignancies after allo-SCT were included in this study, and a total of 34 individual ASED samples were collected and screened. Cyclosporine was detected in 18 ASED samples at concentrations ranging from 6.5–105.0 ng/ml (32.0 ± 22.8 ng/ml, mean ± SD). Mycophenolic acid was found in 19 ASED samples at concentrations ranging from 0.04–25.0 mg/l (4.0 ± 5.4 mg/l, mean ± SD). In 11 batches, both cyclosporine and mycophenolic acid were detected, consistent with concomitant use of these agents. In contrast, everolimus and tacrolimus concentrations were well below the established analytical limits of quantification (< 0.6 and < 0.5 ng/ml) in all ASED samples. In 8 batches, no immunosuppressants could be detected. Distributions of observed mycophenolic acid and cyclosporine ASED concentrations are shown in Fig. 1.

### Clinical observations

#### Visual acuity

**Fig. 2:**
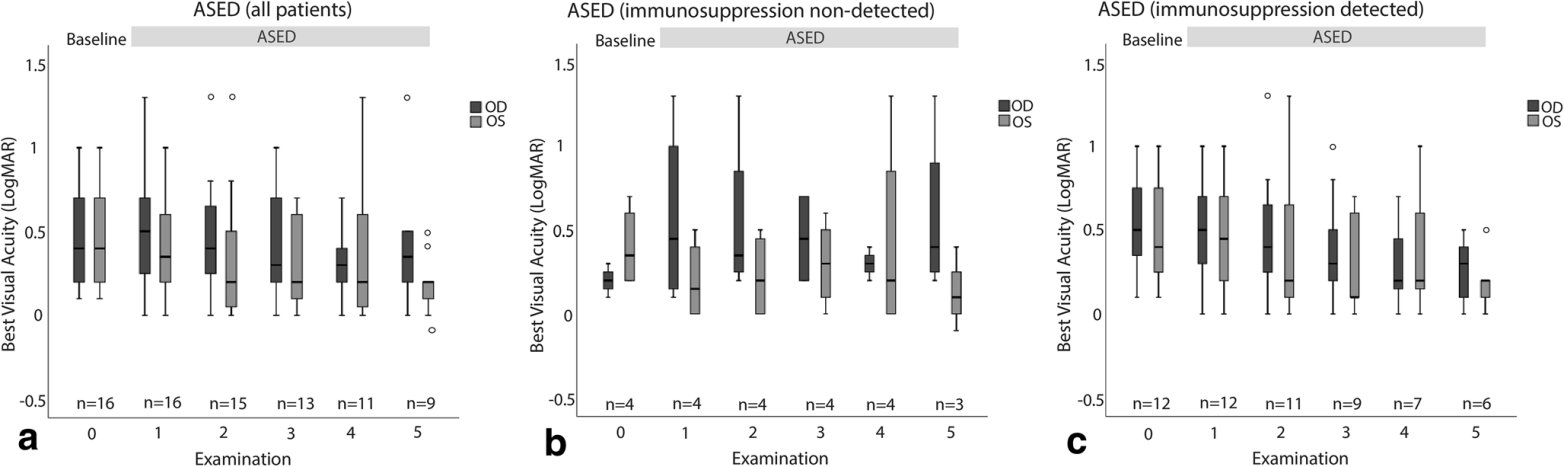
Best corrected visual acuity in logMAR at baseline and follow-up visits under treatment with ASED, divided into analysis of all patients (**a**), patients without detection of immunosuppressants (**b**), and patients with detection of immunosuppressants (**c**). *n*, number of patients included at the respective visit; OD, right eye; OS, left eye; ASED, autologous serum eye drops

Visual acuity was assessed at baseline and on every visit after initiation of therapy with ASED; for every patient, a baseline visit and at least one follow-up visit were available, 15 patients had two follow-up visits, 13 had three follow-up visits, 11 had four follow-up visits, and nine patients were followed for five visits. The results are shown in Fig. 2. Our data shows no significance but a small trend for improvement in visual acuity in both subgroups.

#### Corneal staining

**Fig. 3:**
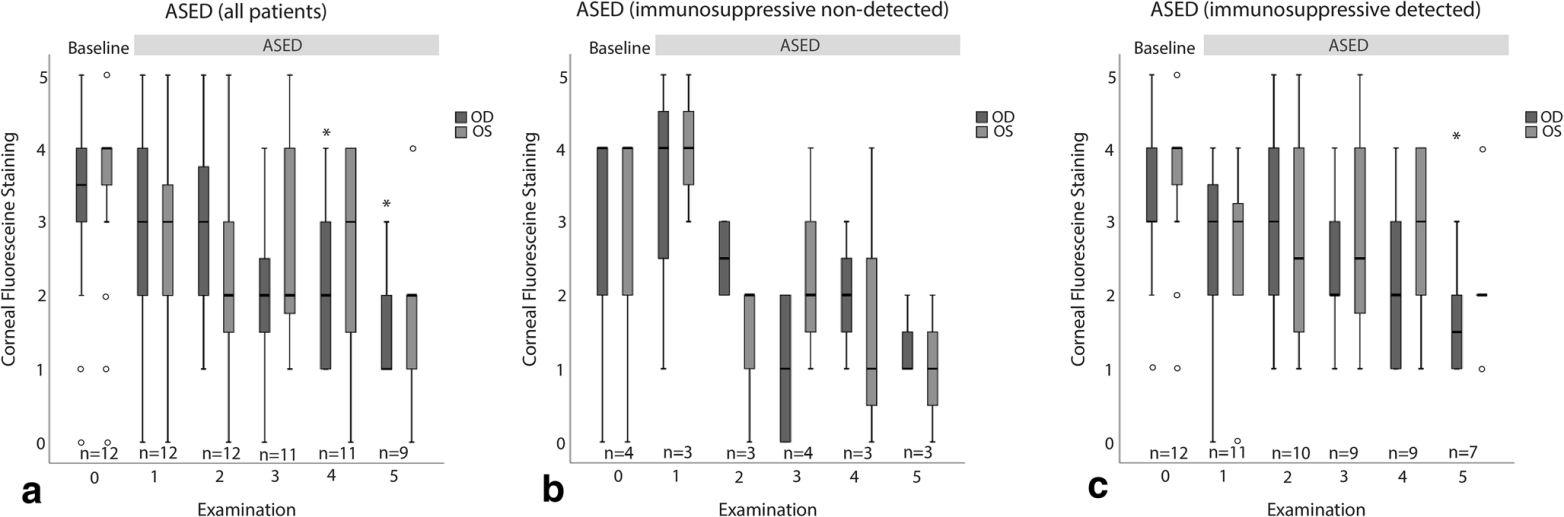
Corneal fluorescein staining at baseline and follow-up visits under treatment with ASED, divided into analysis of all patients (**a**), patients without detection of immunosuppressants (**b**), and patients with detection of immunosuppressants (**c**). *n*, number of patients included at the respective visit; OD, right eye; OS, left eye; ASED, autologous serum eye drops

Corneal fluorescein staining was assessed at baseline and whenever documented at follow-up visits. The results are shown in Fig. 3. A statistically significant improvement is visible at the 4th and 5th examination after the onset of ASED therapy for the right eye in all patients. In the immunosuppressive-positive subgroup analysis, significant improvement was seen at the 5th examination after the onset of ASED therapy.

#### OSDI score

**Fig. 4:**
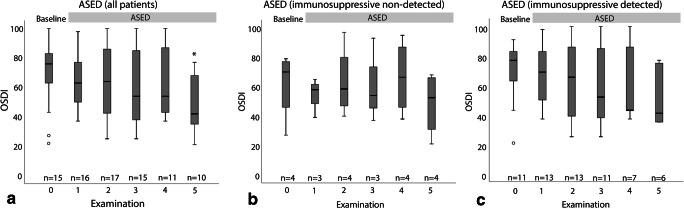
Ocular surface disease index at baseline and follow-up visits under treatment with ASED, divided into analysis of all patients (**a**), patients without detection of immunosuppressants (**b**), and patients with detection of immunosuppressants (**C**). *n*, referring to number of patients included at the respective visit; OD, right eye; OS, left eye; ASED, autologous serum eye drops

The OSDI score [15] was used to quantify subjective discomfort and assess on the baseline and on every visit during ASED therapy, whenever documented. This 15-item questionnaire is routinely used in our outpatient clinic for dry eye patients, as it reliably quantifies symptoms of subjective patient discomfort and visual disturbances, producing a score between 0 (no complaints) and 100 (maximal discomfort). All available OSDI scores were included in statistical analysis and are presented for all patients and for the immunosuppressive-positive and negative subgroups. Significance of improvement is reached at the 5th follow-up examination after the onset of ASED therapy in the analysis of all patients. There is a lack of significance in the group analysis (Fig. 4).

## Discussion

ASED represent a highly effective therapy for many modalities of severe dry eye syndrome of autoimmune, degenerative, or traumatic genesis [17]. They promote wound healing via growth factors like EGF, vitamin A, TGF-beta, and fibronectin, lubricate the ocular surface, lower ocular surface inflammation levels, and are frequently being used to treat patients with severe chronic ocular graft versus host disease and have been shown to be both successful and safe in this entity [17, 18]. However, there are multiple peculiarities regarding this group of patients that may have an impact on therapy with ASED, which have not been sufficiently examined. For one, most patients with ocular GVHD also present with systemic manifestations (e.g., liver, intestines, skin, lung) and need to be treated with systemic glucocorticoids and/or immunosuppressants. In our clinic, the standard regime for patients after hematopoietic stem cell transplantation consists of cyclosporine (in dosages of 150 to 750 mg daily) and mycophenolic acid (in dosages of 500 to 1000 mg daily). Depending on clinical response, this immunosuppressant therapy may be reduced, increased, or changed for tacrolimus or everolimus. Furthermore, antifungal agents are administered following allogeneic stem cell transplantation on a regular basis, since antifungal prophylaxis has been shown to decrease all-cause mortality after chemotherapy, popular substances being amphotericin b, fluconazole, itraconazole, and posaconazole [19]. In CMV-seropositive patients, an additional preemptive therapy with ganciclovir or foscarnet can be necessary [20]. Whether all these systemically applied agents are detectable in ASED was unclear, as well as the implications of the possible presence of pharmacologically active substances in serum eye drops. In this study, we were able to detect systemically applied cyclosporine and mycophenolic acid in ASED manufactured from the blood of patients with severe ocular GVHD for the first time. Since mycophenolic acid does not show relevant distribution between plasma and blood cells [21], it could be detected in ASED at concentrations that are usually achieved with standard doses used for systemic immunosuppression (Fig. 1). In contrast, cyclosporine, tacrolimus, and everolimus are distributed in erythrocytes, and whole blood is the preferred matrix for drug monitoring of these agents [22]. It is well known that cyclosporine levels in whole blood are comparatively higher than in plasma under treatment [23]. In our study, mean cyclosporine ASED concentration was therefore below generally accepted therapeutic target ranges (100–400 ng/ml) [26] which have been established in the whole blood matrix (Fig. 1). In addition, in vitro studies suggested sufficient inhibition of monocytes and lymphocytes by cyclosporine at concentrations ranging from 100 to 500 ng/ml [27], while effective concentrations of mycophenolic acid in vitro were reported to be in the range of 160 ng/ml [28], both well exceeding the concentrations we detected in ASED. Likewise, tacrolimus plasma concentrations can be very low (therapeutic range 5–15 ng/ml) [26] and undetectable by enzyme immunoassay [24], and more than 75% of everolimus is partitioned into red blood cells at therapeutic concentrations [25], which could explain why both substances were not detected in ASED. Concluding from these findings, we are led to suspect that therapy-modifying agents can be found in serum eye drops manufactured from patients receiving systemic therapy in concentrations well below therapeutic ranges in vivo. Moreover, drug concentrations in ASED may likely vary in dependence on the timing of the drug intake relative to the time of blood donation for ASED manufacturing with possibly much higher concentrations of immunosuppressants in ASED when blood is drawn at peak serum concentration. This constitutes a limitation of this study as information was not documented.

Clinical follow-up of our patients did not appear to depict differences of ASED efficacy in regard to detected immunosuppressants at different concentrations, for which we see two possible explanations: either the patient collective was too small to infer the significant impact of systemically administered immunosuppressants on the efficacy of ASED or there is no impact that could be measured. To enhance explanatory power in this important aspect of ASED therapy, it is necessary to observe more patients over longer periods of time to elaborate possible effects of systemic therapy on the efficacy of ASED. Hereby, follow-up periods of up to 9 months are necessary, to be able to detect statistical significant improvements in contrast to clinical observed improvements on signs but most notably symptoms, that often improve only a few weeks after onset of treatment. Since cyclosporine and tacrolimus are being used by ophthalmologists as a topical therapy for GVHD, an additional beneficial effect of ASED containing those substances is possible. To further investigate this hypothesis and to address the second aspect mentioned above regarding possible influence of immunosuppressants on serum eye drops efficacy, we plan to analyze the effects of immunosuppressants on wound healing promotion by ASED in vitro for example using corneal epithelial scratch assays. In the long term, the now established LC-MS/MS method will be used in our clinic to assess drug levels in patients, who show limited or no response to ASED in order to identify possible contraindications for this therapy. This is of clinical relevance, since in recent history there are efforts to provide heterologous serum eye drops for those patients, who are not able to donate blood due to critical general health status. Should a specific systemically applied immunosuppressant be identified as impedimentary to ASED therapy, those patients could directly receive heterologous serum eye drops and fully benefit from this therapy modality. Should, however, a certain immunosuppressive substance show a beneficial effect on ASED therapy, these substances could be added to ASED during the manufacturing process to enhance efficacy, as long as safe and efficient concentrations were to be identified.

## Electronic supplementary material

ESM 1 Exemplary chromatograms for cyclosporine, tacrolimus and everolimus and internal standards of an extracted calibration standard (cyclosporine: 10 ng/ml, tacrolimus and everolimus: 1 ng/ml). Chromatograms for mycophenolic acid are provided in REF 14 (Wiesen et al.). (PNG 231 kb)

High Resolution Image (TIF 645 kb)

ESM 2 Exemplary calibration curves obtained for cyclosporine, tacrolimus and everolimus. (PNG 900 kb)

High Resolution Image (TIF 110 kb)

## References

[1] Ichiki Y, Bowlus CL, Shimoda S (2006). T cell immunity and graft-versus-host disease (GVHD). Autoimmun Rev.

[2] Choi SW, Levine JE, Ferrara JLM (2010). Pathogenesis and management of graft versus host disease. Immunol Allergy Clin N Am.

[3] Jamil MO, Mineishi S (2015). State-of-the-art acute and chronic GVHD treatment. Int J Hematol.

[4] Filipovich AH (2008). Diagnosis and manifestations of chronic graft-versus-host disease. Clin Haematol.

[5] Dietrich-Ntoukas T (2015). Clinical signs of ocular graft-versus-host disease. Klin Monatsbl Augenheilkd.

[6] Engel L, Wittig S, Bock F (2015). Meibography and meibomian gland measurements in ocular graft-versus-host disease. Bone Marrow Transplant.

[7] Heath JD, Acheson JF, Schulenburg WE (1993). Penetrating keratoplasty in severe ocular graft versus host disease. Br J Ophthalmol.

[8] Abud TB, Amparo F, Saboo US (2016). A clinical trial comparing the safety and efficacy of topical tacrolimus versus methylprednisolone in ocular graft-versus-host disease. Ophthalmology.

[9] Inamoto Y, Sun YC, Flowers MED (2015). Bandage soft contact lenses for ocular graft-versus-host disease. Biol Blood Marrow Transplant.

[10] Na KS, Kim MS (2012). Allogeneic serum eye drops for the treatment of dry eye patients with chronic graft-versus-host disease. J Ocul Pharmacol Ther.

[11] Drew VJ, Tseng CL, Seghatchian J (2018). Reflections on dry eye syndrome treatment: therapeutic role of blood products. Front Med.

[12] Ogawa Y, Okamoto S, Mori T (2003). Autologous serum eye drops for the treatment of severe dry eye in patients with chronic graft-versus-host disease. Bone Marrow Transplant.

[13] Tahmaz V, Gehlsen U, Sauerbier L (2017). Treatment of severe chronic ocular graft-versus-host disease using 100% autologous serum eye drops from a sealed manufacturing system: a retrospective cohort study. Br J Ophthalmol.

[14] Wiesen M, Farowski F, Feldkötter M (2012). Liquid chromatography-tandem mass spectrometry method for the quantification of mycophenolic acid and its penolic glucuronide in saliva and plasma using a standardized saliva collection device. J Chromatogr.

[15] Walt JG, Rowe MM, Stern KL (1997). Evaluating the functional impact of dry eye: the ocular surface disease index. Drug Inf J.

[16] Petrescu V, Radojska S, Tahmaz V (2014). Characteristics in manufacturing autologous serum eye drops. Transfusionsmedizin.

[17] Geerling G, Hartwig D (2006) Autologous serum eye drops for ocular surface disorders. Cornea and external eye disease 1–20 10.1136/bjo.2004.044347 PMC1772389 15489495

[18] Tahmaz V, Radojska S, Cursiefen C et al (2017) Autologe Serumaugentropfen – Indikation, Herstellung, Anwendung. Augenheilkunde up2date;6:1–5

[19] Robenshtok E, Gafter-Gvili A, Goldberg E (2007). Antifungal prophylaxis in cancer patients after chemotherapy or hematopoietic stem-cell transplantation: systematic review and meta-analysis. J Clin Oncol.

[20] Reusser P, Einsele H, Lee J (2002). Randomized multicenter trial of foscarnet versus ganciclovir for preemptive therapy of cytomegalovirus infection after allogeneic stem cell transplantation. Blood.

[21] Langman LJ, LeGatt DF, Yatscoff RW (1994). Blood distribution of mycophenolic acid. Ther Drug Monit.

[22] Oellerich M, Dasgupta A (2016). Personalized immunosuppression in transplantation: role of biomarker monitoring and therapeutic drug monitoring.

[23] Mockli G, Kabra PM, Kurtz TW (1990). Laboratory monitoring of cyclosporine levels: guidelines for the dermatologist. J Am Acad Dermatol.

[24] Winkler M, Ringe B, Baumann J (1994). Plasma vs whole blood for therapeutic drug monitoring of patients receiving FK 506 for immunosuppression. Clin Chem.

[25] Kirchner G, Meier-Wiedenbach I, Manns MP (2004). Clinical pharmacokinetics of everolimus. Clin Pharmacokinet.

[26] Dasgupta A (2012). Therapeutic drug monitoring – newer drugs and biomarkers.

[27] Andersson J, Nagy S, Growth CG (1992). Effects of FK 506 and cyclosporin A on cytokine production studied in vitro at single-cell level. Immunology.

[28] Fernandes-Ramos AA, Machetti-Laurent C, Poindessous V et al (2017) A comprehensive characterization of the impact of mycophenolic acid on the metabolism of Jurkat T cells. Sci Rep 7 Article number: 10550 10.1038/s41598-017-10338-6 PMC5585210 28874730

