# Supplementary figures and images for: Detection of systemic immunosuppressants in autologous serum eye drops (ASED) in patients with severe chronic ocular graft versus host disease
Source: Graefes Arch Clin Exp Ophthalmol. 2020 Aug 19;259(1):121–8. doi: 10.1007/s00417-020-04865-8 (PMC7790777; doi:10.1007/s00417-020-04865-8)

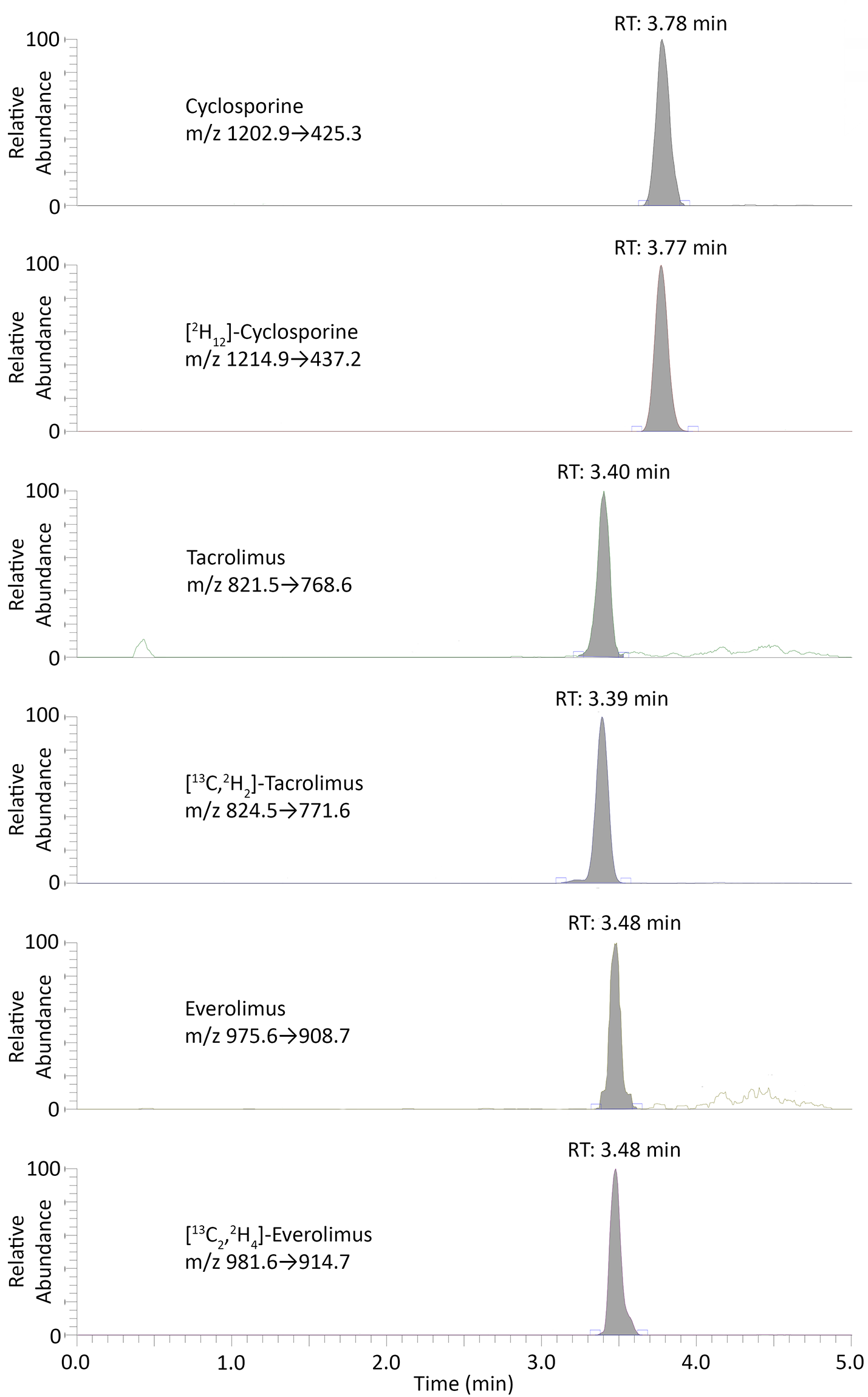

Supplement: Supplementary file 1 — Exemplary chromatograms for cyclosporine, tacrolimus and everolimus and internal standards of an extracted calibration standard (cyclosporine: 10 ng/ml, tacrolimus and everolimus: 1 ng/ml). Chromatograms for mycophenolic acid are provided in REF 14 (Wiesen et al.). (PNG 231 kb) [file 417_2020_4865_Fig5_ESM.png]

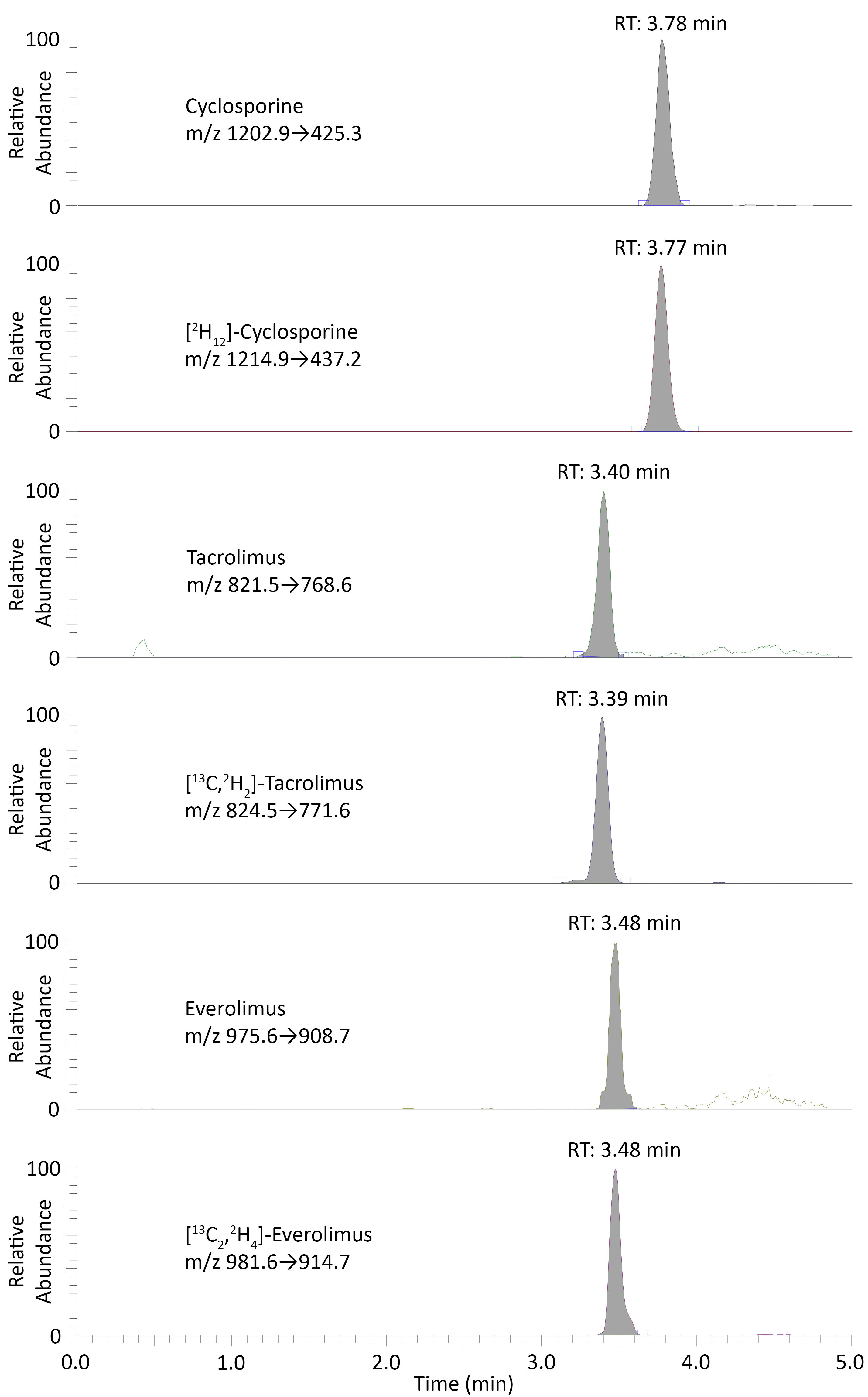

Supplement: Supplementary file 2 — High Resolution Image (TIF 645 kb) [file 417_2020_4865_MOESM1_ESM.tif]

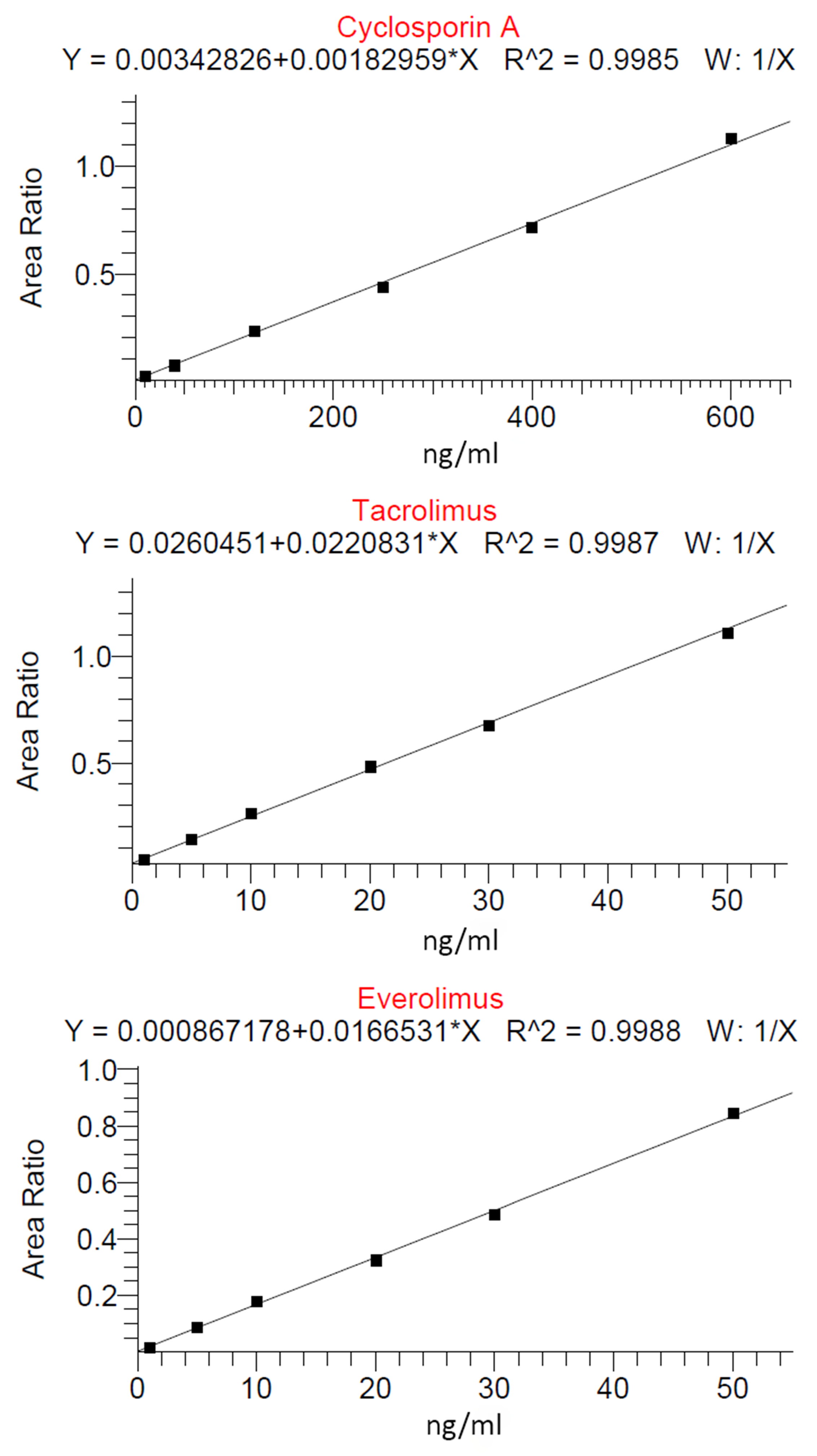

Supplement: Supplementary file 3 — Exemplary calibration curves obtained for cyclosporine, tacrolimus and everolimus. (PNG 900 kb) [file 417_2020_4865_Fig6_ESM.png]

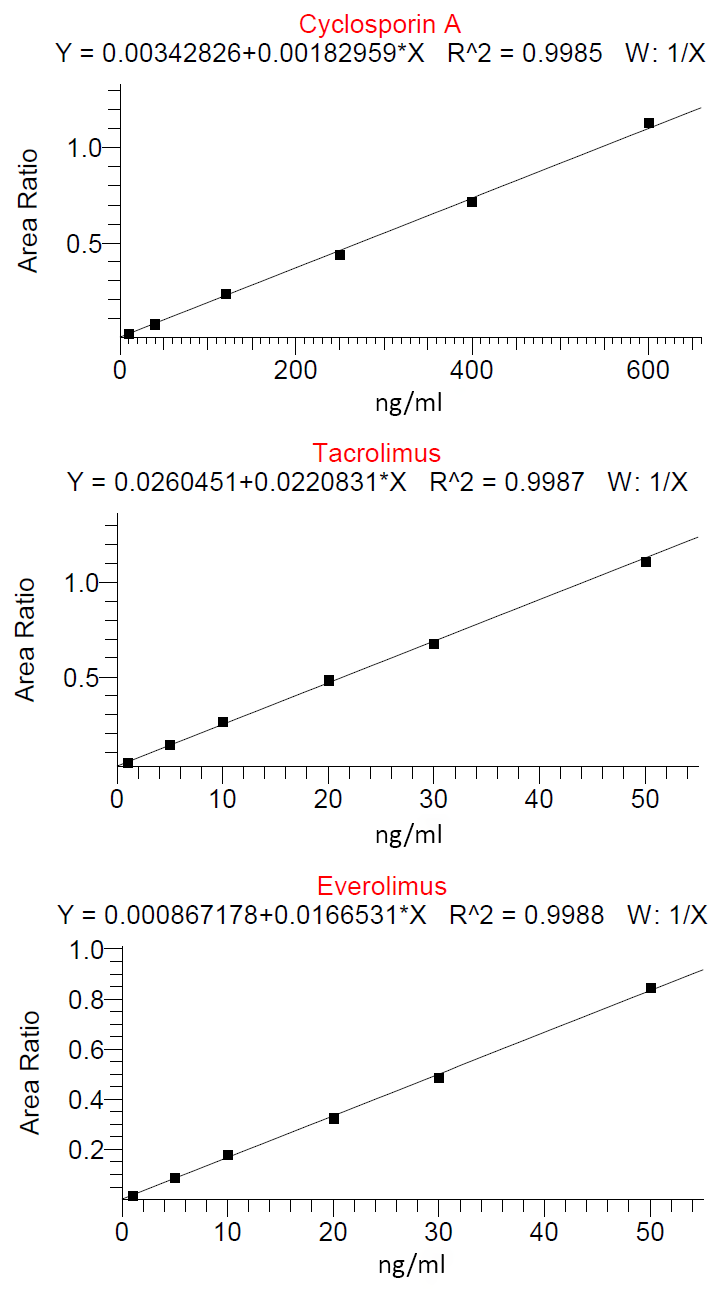

Supplement: Supplementary file 4 — High Resolution Image (TIF 110 kb) [file 417_2020_4865_MOESM2_ESM.tif]
